# Dual roles of methylglyoxal in cancer

**DOI:** 10.3389/fonc.2025.1557162

**Published:** 2025-04-25

**Authors:** Zongao Wang, Shaojun Liu, Minghui Zhang, Min Liu

**Affiliations:** Department of Oncology, Suzhou TCM Hospital Affiliated to Nanjing University of Chinese Medicine, Suzhou, China

**Keywords:** cancer, hormesis, methylglyoxal, glyoxalase 1, therapeutics

## Abstract

Cancer treatment currently includes a variety of approaches. Chemotherapy, targeted therapy, and immunotherapy are combined based on cancer characteristics to develop personalized treatment plans. However, drug resistance can hinder the progress of treatment over time. Methylglyoxal (MG) is a metabolite with hormesis, exhibiting both pro-tumor and anti-tumor actions depending on its concentration during cancer progression. The MG-related metabolic pathway is being explored in the development of anti-cancer drugs, focusing on reducing MG stress or exploiting its cytotoxic effects to inhibit cancer progression. This article investigates the dual role of MG in cancer, emphasizing its effects on cell metabolism and tumor progression. It proposes MG capture therapy for the pre-cancerous stage and MG toxicity therapy for the cancer stage, contributing to the development of precise and individualized cancer treatments.

## Graphical abstract

InSR, Insulin Receptor; NK cell, natural killer cell.

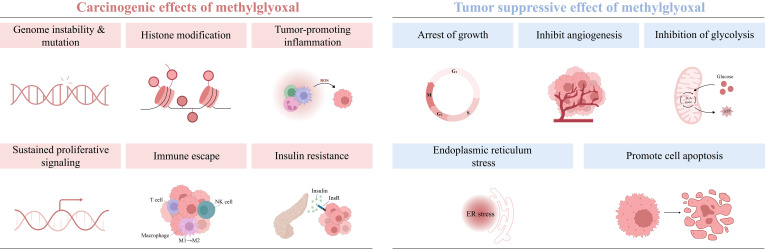

## 1 Introduction

According to data from the International Agency for Research on Cancer (IARC), the incidence and mortality of cancer remain remarkably high. Nearly 20 million new cases and approximately 10 million deaths were reported in 2022; this upward trend is projected to persist (1). Despite significant advances in precision medicine and immunotherapy in recent years, cancer remains a substantial global health challenge (2). Critical risk factors, including smoking, obesity, radiation, and infection, can disrupt normal cellular metabolism, leading to an imbalance between metabolite generation and detoxification, which consequently promotes cancer development. At present, the development of traditional cytotoxic drugs has slowed down (3). At the same time, due to the complexity of cancer cell metabolism and compensatory mechanisms, the efficacy of strategies exclusively targeting tumor cell building block synthesis remains limited (4). Therefore, there is an urgent need to explore new therapeutic targets in order to overcome the limitations of conventional treatments and to enhance the clinical efficacy of cancer therapy.

An important metabolic feature of cancer is the Warburg effect, whereby cancer cells rely predominantly on glycolysis for energy even under normoxic conditions. This metabolic reprogramming not only fulfills the energy demands required for the rapid proliferation of cancer cells but also results in the accumulation of numerous toxic by-products (e.g., methylglyoxal [MG], L-2-hydroxyglutaric acid, and glutaryl coenzyme A) (4–6). MG, a highly reactive dicarbonyl compound produced by glycolysis, has been shown to be strongly associated with a variety of metabolic disorders, including cancer, particularly among high-risk groups such as patients with diabetes mellitus, obesity, or advanced age. Persistent accumulation of MG significantly increases cancer risk (7–9). The abnormal accumulation of MG induces genotoxicity, cellular dysfunction, and subsequent disease by forming advanced glycation end products (AGEs) through glycation modifications of biological macromolecules, including DNA and proteins; this phenomenon is termed “MG stress” (10). Additionally, AGEs further exacerbate inflammation and oxidative stress by activating the receptor for advanced glycation end-products (RAGE), thus promoting tumor growth, invasion, and metastasis (11). In recent years, increasing evidence has indicated that the dysregulation of MG metabolism, particularly abnormal changes in expression levels of the key MG detoxification enzyme glyoxalase 1 (GLO1), is closely associated with the development of various malignant tumors (for detailed mechanisms, see Section 5.2.1).

It is worth noting that the effect of MG exhibits a prominent dose-dependent dual effect known as hormesis: at low concentrations, MG can induce malignant transformation of normal cells and promote carcinogenesis; however, at higher concentrations, it exerts significant cytotoxicity, directly inducing apoptosis in cancer cells and inhibiting tumor growth (12). This unique dose-dependent feature provides important clinical insights into cancer treatment, emphasizing the potential therapeutic value of targeting the MG metabolic pathway. On the one hand, for high-risk groups such as individuals with metabolic syndrome, advanced age or diabetes, lowering MG levels using MG scavengers or GLO1 agonists is expected to be an effective cancer prevention strategy; on the other hand, for cancer cells with high glycolytic activity and high GLO1 expression, targeting the MG metabolic pathway to selectively elevate intracellular MG levels beyond the cytotoxic threshold can significantly enhance the sensitivity of cancer cells to chemotherapy, radiotherapy, and even immunotherapy. This strategy not only provides a therapeutic approach for targeting cancer-specific metabolic alterations but also holds promise for overcoming drug resistance and toxic side effects associated with traditional therapies.

Therefore, an in-depth understanding of the dual mechanism of MG metabolism in cancer progression, as well as clarification of the precise applications of targeting MG metabolic pathways in cancer prevention and treatment, is of substantial clinical significance and research importance. The aim of this review is to summarize the latest advances in research regarding MG metabolism and cancer development, to propose specific directions for future investigation and potential clinical strategies, and to provide a novel theoretical basis and practical guidance for precision cancer therapy.

## 2 The biological processes of MG

### 2.1 Formation of MG

MG is a highly reactive dicarbonyl compound with cytotoxic properties and has the molecular formula C3H4O2. It was first identified in red blood cells. Several studies have reported on the concentration of MG under physiological conditions, but significant discrepancies exist across the studies. For instance, Rabbani et al. reported the concentration range of MG in mammalian cells as 1–4 mM using stable isotope dilution liquid chromatography-tandem mass spectrometry (SID-LC-MS/MS), whereas the plasma MG level in healthy individuals was measured at 132 ± 63 nM (13). In contrast, plasma MG concentrations in healthy individuals measured by Scheijen et al. were higher, reaching 212 ± 8 nM (14). This significant discrepancy in measurements may stem from variations in assay methodology, sample preparation, instrument sensitivity, and study sample size. Specifically, the higher concentrations reported by Scheijen et al. may be attributed to their relatively small sample sizes or insufficient elimination of interfering factors during the experimental procedure, whereas the study by Rabbani et al. may reflect a more rigorous sample pretreatment and analytical approach. However, few studies in the literature systematically compare different assays, highlighting the urgent need for larger, standardized studies to clarify baseline MG levels across various tissues and physiological states. Furthermore, clarifying the reasons for differences in measurements is essential to elucidate the pathologic links between MG levels and disease such as diabetes and cancer.

**Figure 1:**
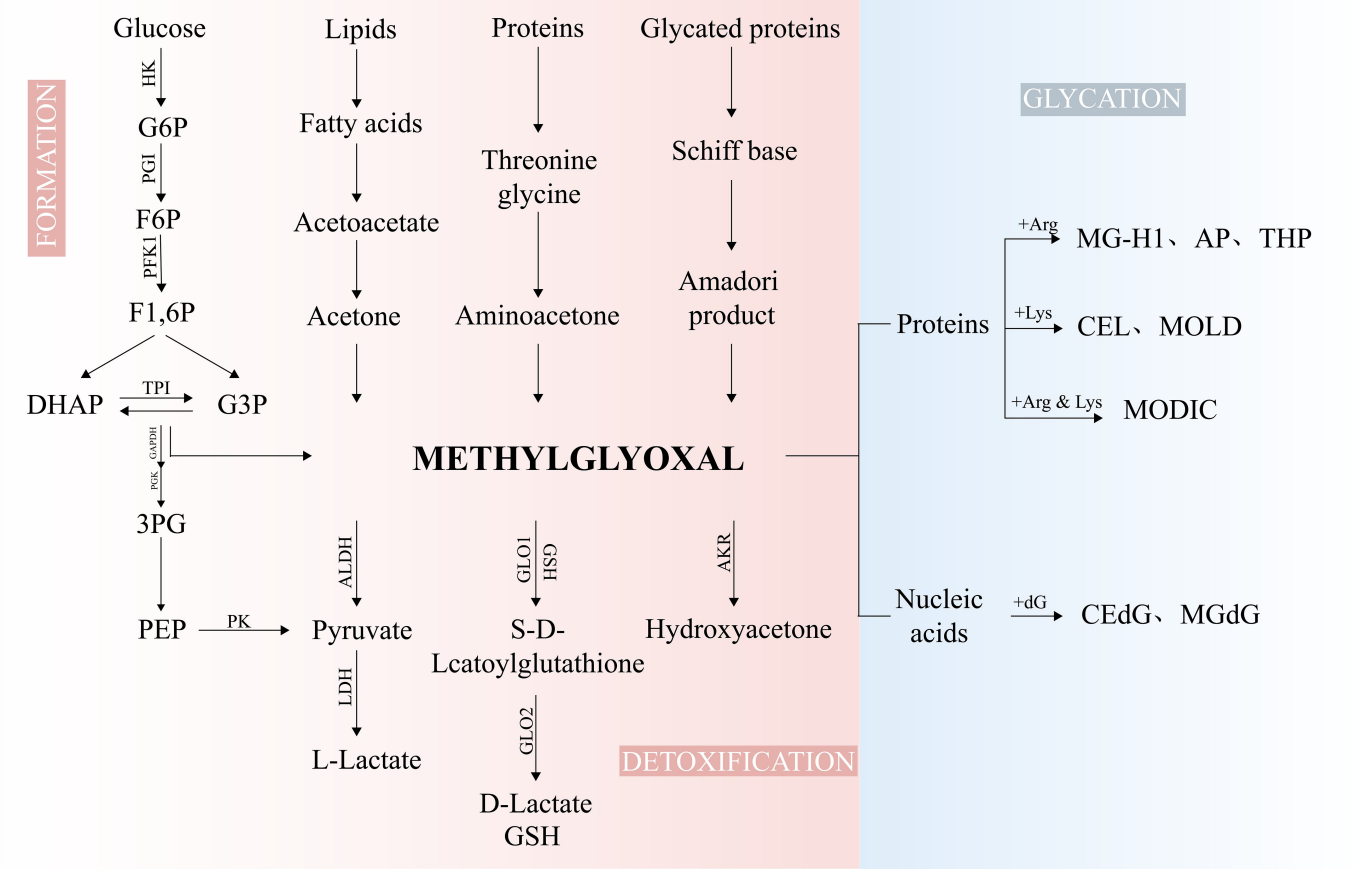
The biological processes of MG. Glycolysis, serving as the major source of MG, is generated by non-enzymatic reactions of TPI, DHAP, and G3P. The minor sources of MG arise from the metabolism of proteins, lipids, and glycated proteins. MG is metabolized into D-Lactate, pyruvate, and hydroxyacetone under the action of GLO1/2, ALDHs, and AKRs. MG can modify DNA and proteins to form AGEs. F6P, fructose-6-phosphate; G6P, glucose-6-phosphate; F1,6P, fructose-1,6-bisphosphate; DHAP, dihydroxyacetone-phosphate; G3P, glyceraldehyde-3-phosphate; TPI, Triosephosphate isomerase; 3PG, 3-phosphoglycerate; PEP, phosphoenolpyruvate; HK, hexokinase; PGI, phosphoglucose isomerase; PFK1, phosphofructokinase-1; GAPDH, glyceraldehyde-3-phosphate dehydrogenase; PGK, phosphoglycerate Kinase; PK, pyruvate kinase; LDH, lactate dehydrogenase; CEdG, N2-carboxyethyl-2’-deoxyguanosine; MGdG, 3-(2’-deoxyribosyl)-6,7-dihydro-6,7-dihydroxy-6/7-methylimidazo-[2,3-b]purine-9(8)one; MG-H1, methylglyoxal-derived hydroimidazolone 1; Argpyrimidine/AP, N-delta-(5-hydroxy-4,6-dimethylpyrimidine-2-yl)-L-ornithine); CEL, Nϵ-carboxyethyllysine; MOLD, 1,3-di(Nϵ-lysino)-4-methyl-imidazolium; MODIC, 2-ammonio-6-{(2-[(4-ammonio-5-oxido-5-oxopentyl)amino]-4-methyl-4,5-dihydro-1H-imidazol-5-ylidene)amino}hexanoate.

The major pathways of MG production include glycolysis (9), glycated protein degradation, lipid, carbohydrate, and protein metabolic processes (15, 16) (**Figure 1**). Additionally, dietary intake constitutes one of the primary sources of MG in the human body (17). Consequently, plasma MG concentrations are generally higher in diabetic patients than in healthy individuals, with a reported mean value of 277 ± 9 nM (14). Under normal circumstances, the formation of MG accounts for only 0.1 - 0.4% of the glucose flux in glycolysis (18), yet its glycation activity is 10,000 - 50,000 times that of glucose and is regarded as the most potent glycating agent (19). Abnormal accumulation of MG is capable of triggering MG stress, which has been linked to the pathology of various diseases, including aging, diabetic complications, and cancer (10).

### 2.2 Detoxification of MG

MG, as a cytotoxic metabolite, can cause severe cellular damage if accumulated in excess. In response to this potential threat, specialized intracellular detoxification mechanisms exist to prevent MG accumulation. Detoxification of MG mainly depends on the glyoxalase system. This system was first discovered by Dakin-Dudley and Neuberg et al. in 1913 (20) and consists of GLO1, GLO2, and glutathione (GSH), which are widely present in the cytoplasm of mammalian cells, with GLO2 is also partially distributed in mitochondria (21). The detoxification process of the glyoxalase system depends on GSH: MG first reacts nonenzymatically with GSH to produce a hemisulfide acetal intermediate, which is subsequently converted to S-D-lactoylglutathione catalyzed by GLO1; immediately thereafter, S-D-lactoylglutathione is hydrolyzed to the nontoxic D-lactic acid catalyzed by GLO2 and releases GSH to reenter the next cycle. Ordinarily, the glyoxalase system metabolizes 99% of MG, safeguarding the proteome and genome from damage (22). Therefore, the glyoxalase system plays a vital role throughout the processes of cell development, maturation, aging, and death, and is essential for maintaining normal biological functions (23). Notably, aldehyde dehydrogenases (ALDHs) and aldehyde-keto reductases (AKRs) can convert MG to pyruvate or hydroxyacetone, respectively, undertaking a small amount of MG detoxification (24, 25).

### 2.3 The glycation reaction of MG

Glycation is a common non-enzymatic biochemical reaction in which the carbonyl group on a reducing sugar molecule reacts with the amino group of a protein, nucleic acid, or lipid to form AGEs, a process also known as the Maillard reaction (26). MG, an important precursor of AGEs, exerts strong pro-oxidative and pro-inflammatory effects, inducing protein and DNA dysfunction through glycation modifications, thereby triggering pathological responses such as cellular dysfunction (7, 10). MG modifies DNA through non-enzymatic reactions, with deoxyguanosine (dG) being the most susceptible nucleotide. Common MG-DNA adducts, including CEdG and MGdG, were identified using SID-LC-MS/MS,. These MG-DNA adducts contribute to genomic instability, exhibit mutagenic potential, and are associated with malignant transformation (27). MG also interacts with proteins by modifying amino acid residues, primarily arginine and lysine, thereby altering their structure and function. The protein adducts induced by MG include MG-H1, AP, and CEL (28, 29). Specific arginine residues, such as Arg-114, Arg-186 and Arg-218, have been identified as key sites for MG modification (30). Amino acid modification by MG significantly alters the structure and function of proteins, which in turn affects cell signaling, including the induction of apoptosis and the regulation of epithelial-mesenchymal transition (EMT) processes. This alteration is closely associated with various pathological processes such as aging, diabetes, cardiovascular disease, and cancer (31, 32). Notably, Aglago et al. measured and analyzed plasma AGEs in 1,378 patients with primary colorectal cancer using ultra-high-performance liquid chromatography-tandem mass spectrometry (UHPLC-MS/MS). Their findings revealed a strong association between methylglyoxal-derived AGEs (CEL, MG-H1) and the risk of colorectal cancer (33). The aforementioned studies suggest that MG-mediated glycation plays a crucial role in the pathological process of cancer.

## 3 MG mediates the occurrence and development of tumors

**Figure 2:**
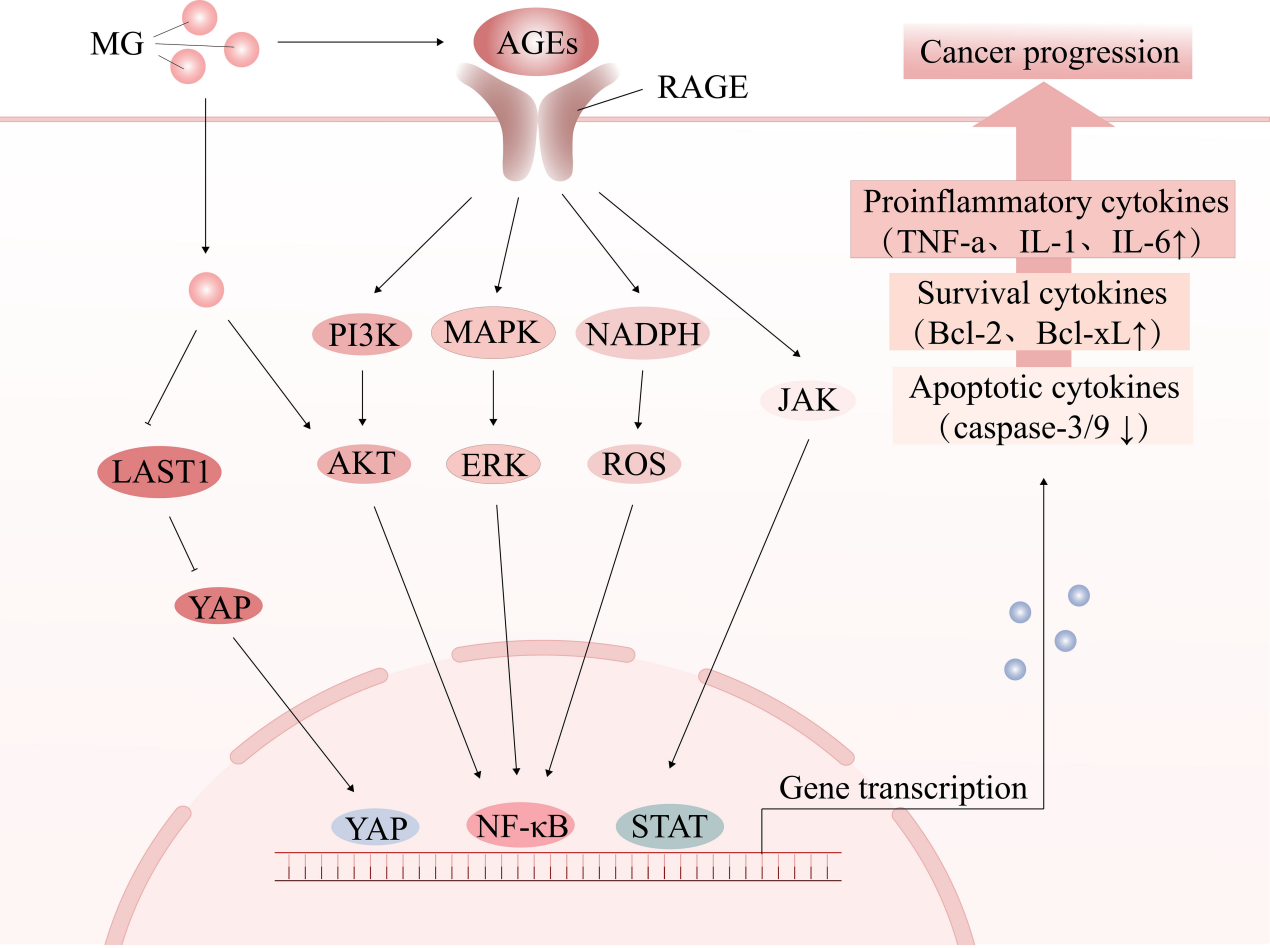
The tumor-promoting effect of MG. MG promotes oxidative stress and chronic inflammation by activating the AGE-RAGE signaling pathway or directly modifying signaling molecules, thereby creating a microenvironment conducive to cancer development, influencing cell proliferation and apoptosis, and facilitating cancer progression.

Tumorigenesis and progression are driven by on complex interactions between cancer cells and their microenvironment. The tumor microenvironment (TME) consists of immune cells, cancer-associated fibroblasts (CAFs), the vascular system, the extracellular matrix (ECM), and a metabolic microenvironment characterized by inflammation and oxidative stress. MG, a highly reactive metabolite, significantly impacts cancer cell signaling and the TME by modifying nucleic acids, proteins, and lipids, thereby promoting tumor development. (**Figure 2**) (**Table 1**). Early studies have shown that continuous subcutaneous injection of MG (0.2 mL/dose, 10 mg/mL twice a week for 10 weeks) induced tumor formation in rats, with a tumorigenicity rate of 22.22% (34). In colorectal cancer (CRC) and anaplastic thyroid cancer (ATC) tissues, MG adduct levels adducts are positively correlated with tumor malignancy and metabolic activity, and the oncogenic effects of MG can be effectively reversed by MG scavengers or GLO1 agonists (32, 35). Epidemiological studies further support the association between MG accumulation and increased cancer risk, particularly in populations with metabolic abnormalities such as advanced age, obesity, and hyperglycemia (7, 36, 37).

**Table 1:** The pro-cancer manifestations of MG/AGEs *in vivo* and *in vitro*.

*In vitro*				
Cancer Categories	Cell Types	Processing approach	Laboratory Findings	References
ATC	AL62	MG: 5uM	Migration and invasion capacity↑	(32)
SqCLC	SW1573	IHC	▲ AP-Hsp27↑. Caspase↓ ▲ Evade cell apoptosis	(72)
NSCLC	NCI-H231	IHC		(71)
PC	T3M4	The Gemcitabine Provocation Test	▲ Glycolysis (GLUT1 and LDH) and MG stress (AP)↑ ▲ Acquiring gemcitabine resistance	(95)
	MIA-PaCa-2	MG: 200uM; CEL: 100ug/mL	▲ YAP↑ ▲ cell proliferation↑	(75)
BC	MDA-MB-231; MDA-MB-468	shGLO1; MG: 300/500uM	▲ MEK/ERK/SMAD1↑ ▲ Migration and invasion capacity↑	(77)
	MCF-7	MG-BSA-AGEs: 50/100 ug/ml	▲ p-ERK1/2, p-CREB1↑ ▲ Proliferation and migration ability↑	(78)
	MDA-MB-231	MG-BSA-AGEs: 50/100 ug/ml	▲ MMP9, p-ERK1/2, p-STAT3↑ ▲ Proliferation, migration, and invasive ability↑	(79)
	MDA-MB-231; MDA-MB-468	MG: 300uM	▲ YAP, vimentin↑. E-cadherin↓ ▲ Proliferation and migration ability↑	(74)
CC	SW480	AGEs: 100ug/ml	▲ PI3K/Akt↑ ▲ Cell proliferation, invasion, and EMT↑	(76)
CRC tissue		IHC	The high expression of AP and MG-H1 is positively associated with the malignancy and metabolic activity of the tumor.	(35)
CRC	KRAS^WT-LIM121	shGLO1	▲ MG stress, Akt↑ ▲ Acquiring resistance to cetuximab	(96)
CC	HCT116	AGEs: 50-400mg/l	▲ ChREBP↑ ▲ Cell proliferation (max: 200mg/l)↑	(94)
HCC	HepG2			
*In vivo*				
Cancer Categories	Model	Processing approach	Laboratory Findings	References
BC	mice/chicken embryo membranes	shGLO1-MD-MBA-231	▲ Nuclear accumulation of YAP↑ ▲ Tumorigenic and metastatic capacity↑	(74)
	mice	shGLO1-MD-MBA-231	Pulmonary tumor burden↑	(77)
PC	KC-Mito mice	Carnosine (FL-926-16): 30mg/kg/day, Oral	Mice untreated with carnosine exhibited a higher incidence of pancreatic cancer.	(75)

SqCLC, squamous cell lung cancer; NSCLC, non-small cell lung cancer; PC, pancreatic carcinoma; BC, breast cancer; HCC, hepatocellular carcinoma; CC, colon cancer; IHC, immunohistochemistry.

↑: High expression/promotion; ↓: Low expression/inhibition.

### 3.1 Gene mutagenesis

Cancer development typically involves activation of proto-oncogenes and inactivation of tumor suppressor genes, with these alterations are closely associated with DNA damage and mutation accumulation. In addition to traditional oncogenic factors such as radiation, inflammation, and aging, MGs also exhibits significant genotoxicity (38, 39). MG modifies approximately 1% of DNA nucleotides, increasing the frequency of base mismatches, particularly at the G:C site (40, 41), and inducing various forms of DNA damage, such as sister chromatid exchanges (42), DNA adduct formation (43), and strand breaks (27). Additionally, MG induces the degradation of the tumor suppressor protein BRCA2, weakening the cell’s DNA repair capacity and further increases mutation accumulation (44, 45). Despite the cell’s defense mechanisms against DNA glycation, the long-term accumulation of MG eventually exceeds the cell’s repair capacity, causing a significant increase in mutational load. Therefore, studying MG-mediated DNA modification can help to elucidate the mechanism of mutation, which is particularly critical for cancer prevention in individuals with advanced age or metabolic disorders.

### 3.2 Histone modification

Histone modifications play a central role in maintaining chromosome structure and regulating gene expression, serving as an important modality for epigenetic regulation (46). MG primarily modifies arginine and lysine residues in the N-terminal tail of histones, affecting their structure and function, decreasing nucleosome stability, leading to chromatin loosening, and thus altering gene transcriptional activity (47). Simultaneously, MG modifications can also alter the electrically charged properties of histones, thereby weakening their binding strength to DNA, affecting the DNA-binding efficiency of transcription factors, and activating or inhibiting the expression of genes associated with carcinogenesis (48). Additionally, MG modifications may reduce the interaction efficiency of histones with DNA damage repair proteins, leading to DNA damage accumulation and further increasing the risk of tumorigenesis (49). Moreover, MG modifications may have synergistic or competitive effects with other histone modifications, such as acetylation and methylation, to co-regulate the expression of tumor-associated genes (49, 50). Studies have shown that high levels of histone MG modifications in breast cancer cells are associated with abnormal cell proliferation (51). Additionally, MG-specific modifications of histones may trigger autoimmune responses, making MG-modified histone autoantibodies promising novel immune markers for early diagnosis and prognostic assessment of cancer (52). Thus, MG profoundly impacts epigenetic regulation through histone glycation modification, offering a new dimension of understanding in tumorigenesis and development.

### 3.3 The cross-linking of MG stress, oxidative stress and chronic inflammation

One of the key features of MG stress is the activation of RAGE, which triggers a series of signaling cascades that drive chronic inflammation and oxidative stress, thereby promoting tumor progression (53).

#### 3.3.1 MG stress and oxidative stress

Imbalance in redox homeostasis is one of the hallmarks of cancer cells and can lead to malignant tumor progression and drug resistance (54). MG activates various transcription factors, such as MAPK, NF-κB, and p-21, through the AGEs/RAGE pathway (55), and promotes NADPH oxidase activation, causing ROS accumulation and oxidative stress (56–58). Furthermore, MG further increases ROS production through RAGE-independent pathways, such as mitochondrial damage (59), inhibition of antioxidant enzymes (60), and non-enzymatic reactions (57), exacerbating oxidative damage and mutation risk. Notably, oxidative stress can drive a shift in cellular metabolic patterns from oxidative phosphorylation (OXPHOS) to glycolysis, a process known as the Warburg effect. For example, ROS cause a decrease in OXPHOS efficiency by affecting mitochondrial DNA (mtDNA), electron transport chain (ETC) complexes (e.g., complexes I and III), and membrane lipids, which prompts the cell to shift to glycolysis to maintain energy metabolism (61). It has been shown in CAF that accumulation of ROS leads to mitochondrial dysfunction and a shift toward glycolytic metabolism, thus creating a nutrient-rich microenvironment to meet the metabolic demands of cancer cells (62). Meanwhile, oxidative stress prompts isolated cancer cells to aggregate and form cell clusters, inducing microenvironmental hypoxia, thereby activating hypoxia-inducible factor 1-α (HIF-1α)-mediated glycolysis (63). The increase in glycolytic activity is accompanied by an increase in MG production, thus forming an oxidative stress-MG stress feedback loop. However, there is no direct evidence to support the establishment of the “oxidative stress-glycolysis-MG stress” loop, but it provides an important direction for future research.

#### 3.3.2 MG stress and chronic inflammation

The role of chronic inflammation in cancer progression is well-established (64). Circulating AGEs levels have been found to correlate with the inflammatory marker C-reactive protein (65). Activation of relevant transcriptional regulators (NF-κb, STAT3, HIF-1α) by the MG/AGEs/RAGE axis promotes increased secretion of inflammation-associated factors (e.g., TNF-α, IL-1, IL-6) (11, 65), as well as the recruitment of inflammatory cells, thereby shaping an inflammatory microenvironment that promotes tumor growth (66). Additionally, foodborne MG also promotes the release of pro-inflammatory factors such as IL-8 and IL-6 (67). In turn, chronic inflammation can also inhibit GLO1 activity, leading to further accumulation of MG, thus creating a vicious cycle that promotes cancer development, thus forming a closed loop. The link between chronic inflammation and cancer driven by MG/RAGE signaling has been discussed in detail (11) and will not be revisited here.

The crosstalk between chronic inflammation and oxidative stress has been well-established (68). In conclusion, the crosstalk between MG stress, oxidative stress and chronic inflammation has become a reality, and MG acts as a bridge to construct the microenvironment of tumorigenesis and development.

### 3.4 Cellular proliferation and apoptosis

One of the important mechanisms by which MG promotes tumor growth is through the regulation of signaling pathways involved in cell proliferation and apoptosis. MG directly modifies the Cys(77) site of Akt, increasing Akt phosphorylation, and promote cell cycle progression and cell proliferation (69, 70). Additionally, MG inhibits cytochrome c-mediated caspase activation and blocks apoptosis by modifying Hsp27. Studies have shown that MG modification of Hsp27 significantly reduces the sensitivity of tumor cells to chemotherapeutic drugs, a phenomenon closely associated with the activation of the Akt pathway (71–73). Furthermore, MG can inhibit the activity of large tumor suppressor 1 (LATS1) and promote the translocation of YAP to the nucleus by modifying Hsp90, accelerating the proliferation and metastasis of breast cancer cells (74). In a diabetes-associated pancreatic cancer model, MG significantly promotes the development and invasion of KrasG12D/+ pancreatic cancer through activation of ERK1/2 signaling and YAP nuclear translocation (75). In ATC cells, MG similarly promotes TGF-β1 secretion and activates the FAK signaling pathway, thereby enhancing their invasive phenotype (32).

AGEs formed by MG also regulate the proliferation and migration of tumor cells through the RAGE signaling pathway. For example, AGEs can activate the PI3K/Akt signaling pathway, inhibit apoptosis of colon cancer cells, and promote their proliferation and metastasis (76). In breast cancer cells, low-dose AGEs activate ERK1/2 and CREB1 to enhance cancer cell proliferation and migration (77, 78), and similar RAGE-dependent pro-proliferative and migratory effects were observed in the highly invasive breast cancer MDA-MB-231 cell line (79).

In summary, MG plays a key role in cancer cell survival and proliferation by regulating multiple signaling pathways, including Akt, Hsp27, and YAP, and modulating cell proliferation and anti-apoptotic signaling through the AGEs/RAGE pathway.

### 3.5 Immune escape

The immune system plays a key role in the prevention and treatment of cancer. However, MG can impair the immune response and facilitate immune escape. Natural killer (NK) cells, as important anti-tumor immune cells, are required to kill tumor cells by forming cleavage synaptic clefts to contact tumor cells. However, MG modification of NK cell proteins may interfere with this contact, affecting the recognition and adhesion of NK cells to tumor cells and inhibiting their immunocidal effects on tumor cells without affecting the cells’ own activity (80). Myeloid-derived suppressor cells (MDSCs) are commonly recruited to the tumor microenvironment (81), where they transfer self-produced MG to T cells in a cell-contact-dependent manner, depleting intracellular L-arginine and inducing T-cell paralysis (82). MG also inhibits the expression of CD83, a maturation marker on the surface of dendritic cells (DCs), impeding DC maturation and T cell activation capacity (83); meanwhile, MG induces oxidative damage and apoptosis in human monocytes (84). Furthermore, MG promotes macrophage polarization toward an immunosuppressive M2 phenotype (85, 86), and the above suggests that MG drives cancer cell immune escape by affecting the immune cell’s own function and activity. Notably, MG can also affect the expression of tumor cell-associated immune proteins, thereby interfering with anti-tumor immune effects. For example, MG treatment increased the expression of CD24 protein in breast cancer cells to evade phagocytosis by macrophages (77, 87). Therefore, in the therapeutic strategy, the MG burden is reduced by the MG scavenger N-acetylcysteine (NAC) (88) and genistein attenuates MG stress damage to immune cells (84), which restores anti-tumor immunity and reduces the risk of immune escape.

### 3.6 Insulin resistance

Hyperinsulinemia, a hallmark of insulin resistance (IR), creates a tumor-friendly environment by promoting cellular proliferation (89). Obesity and metabolic syndrome significantly increase MG accumulation, further exacerbating IR and promotes type 2 diabetes (T2D) (90). The exact mechanism of MG-induced IR is still unclear and may involve the following aspects: disrupting the normal conduction of the insulin signaling pathway (91); decreasing the stability of the insulin molecule itself (92); and directly decreasing the sensitivity of insulin-targeted tissues. These mechanisms interact to form a vicious circle, further exacerbating metabolic abnormalities and ultimately promoting the formation of the tumor microenvironment, suggesting that the MG metabolic pathway is an important potential target for cancer prevention and treatment.

Furthermore, MG/AGEs promote the proliferation, invasion, and migration of tumor cells by cross-linking the extracellular matrix (93) and modifying metabolism (94). To summarize, MG constructs a cancer-promoting microenvironment through multilevel modifications; in-depth study of MG metabolic abnormalities will provide important theoretical and clinical insights for cancer prevention and treatment.

## 4 MG inhibits the occurrence and development of tumors

**Figure 3:**
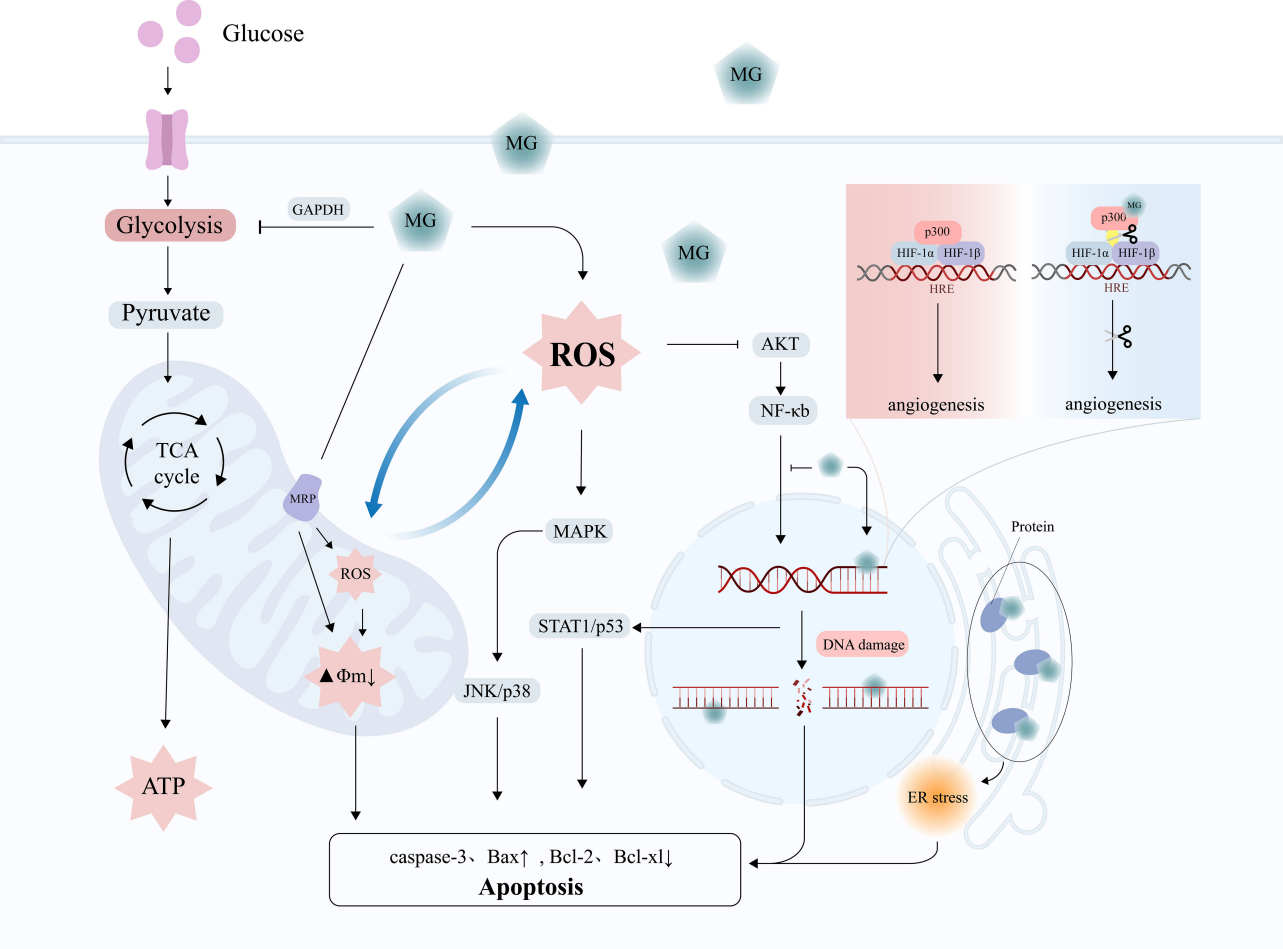
The tumor-suppressive effects of MG. MG exerts anti-tumor effects through mechanisms such as DNA damage, oxidative stress, mitochondrial damage, activation of apoptosis signals, and inhibition of angiogenesis.

In recent years, researchers have identified the dual role of MG in tumors: at lower doses, it induces the malignant transformation of normal cells, resulting in carcinogenesis and invasion; at higher doses, it directly induces cell apoptosis and inhibits tumor growth, a phenomenon known as hormesis (12). MG exerts cytotoxic effects on cancer cells through multiple mechanisms, including inducing cell growth arrest, activating apoptosis/necrosis signals, and inhibiting glycolysis (**Figure 3**) (**Table 2**).

**Table 2:** The tumor-suppressive manifestations of MG *in vivo* and *in vitro*.

*In vitro*				
Cancer Categories	Cell Types	Processing approach	Laboratory Findings	References
Leukemia	HL60	MG: 33-524uM IC50 = 238uM	▲ Inhibit DNA and protein synthesis. ▲ Induce cell growth arrest; ▲ High concentrations induce cell apoptosis.	(31, 101)
	Jukat	MG: 0.25-0.5mM	▲ JNK, caspase-3↑ ▲ Cell apoptosis↑	(114)
CML	Blood	MG: 1/2mM	▲ Mitochondrial complex I, glycolysis, ATP↓ ▲ Cell viability↓	(123)
EAC	EAC cell	MG: 2/5mM	▲ Glycolysis, mitochondrial respiratory, ATP↓ ▲ Tumor cell death↑	(121, 122)
		MG: 0.2/0.4mM	▲ DNA and protein synthesis↓ ▲ Cell proliferation↓	(100)
EAC		MG: 2/5mM	Glycolysis, mitochondrial respiratory↓	(120–123)
Leukemia		MG: 1/2mM		
CESC				
PC	PC-3	MG: 1-5mM	▲ Cyclin D1, CDK2, CDK4↓ ▲ Glycolysis, Cell proliferation↓ ▲ Cell cycle arrest (G1), cell apoptosis↑	(124)
	LNCaP	MG: 1mM	▲ NF-kB, Bcl-2, Bcl-XL↓. Bax↑ ▲ Cell apoptosis↑	(109)
GBM	U87MG/T98G	MG: 25uM	▲ ATP↓ ▲ Cell cycle arrest (G0), cell apoptosis↑	(102)
BC	MDA-MB-231/4T1	MG: IC50: 0.6mM/1.0mM	▲ ROS, caspase, Bax↑ ▲ Bcl-2, p-AKT, nuclear translocation of NF-κB↓ ▲ Cell proliferation↓ Cell apoptosis↑	(110)
	MCF-7	MG: 24h IC50 = 2.7mM 48h IC50 = 1.4mM	Induce mitochondrial functional disorder, suppress the proliferation of tumor cells.	(132)
	MCF-7	MG-BSA-AGEs: 200ug/ml	▲ caspase-3↑ ▲ Cell proliferation↓ Cell apoptosis↑	(78)
	MCF-7/MDA-MB-231	MG: 0.4/0.8uM; shGLO1	▲ p-JNK, p-p38↑ MMP-9, Bcl-2↓ ▲ Cell proliferation, migration, and invasion↓ ▲ Cell apoptosis↑	(133)
Osteosarcoma	U266	MG: 5/10uM+ Cisplatin: 3ug/ml	▲ ROS, PKCδ↑ ▲ Strengthen cisplatin-induced cell apoptosis.	(113)
HCC	Huh-7/HepG2	MG: 1uM	▲ Facilitate the nuclear translocation of p53; ▲ Attenuate the migratory, invasive and adhesive capabilities of cancer cells.	(116)
RC	CT26	MG: 200uM + RT 6Gy	▲ ER stress, cGAS-STING↑ ▲ ROS, Caspase-3, Bax↑ Bcl-2↓ ▲ Enhanced immune cell infiltration within the tumor (CD8+ T cells, NK cells) ▲ Cell apoptosis↑ Tumor volume↓	(131)
CRC	SW480/SW620/DLD-1/HCT-15	shGLO1	▲ STAT1, p53, Bax↑ c-Myc, Bcl-2↓ ▲ Cell apoptosis↑ ▲ Cell proliferation and migration↓	(134)
NSCLC	3553T3/LGSP	MG: 200uM	▲ MG-H1↑ ▲ Cell viability↓	(135)
*In vivo*				
Cancer Categories	Model	Processing approach	Laboratory Findings	References
YOSHIDA ASCITES HEPATOMA	AH-130 mice	MG:12.5,25,50,100mg/kg Intraperitoneal	▲ Cell proliferation↓ ▲ Cell cycle arrest (S)↑ ▲ Survival rate and survival period of tumor-bearing rats↑	(99)
BC	4T1 mice	MG:15, 35mg/kg tail vein injection	▲ Cell apoptosis↑ ▲ Tumor growth↓	(110)
CRC	shGLO1-SW620mice		▲ Tumor growth↓	(134)

CML, chronic myelocytic leukemia; EAC, Ehrlich ascites carcinoma; CESC, cervical squamous cell carcinoma; GBM, glioblastoma; RC, rectal cancer.

↑: High expression/promotion; ↓: Low expression/inhibition.

### 4.1 Growth arrest

The anti-cancer potential of MG was first proposed in the 1960s (97). Early studies have shown that intraperitoneal injection of MG in mice inhibits the growth of ascites sarcoma 180 cells but has limited effect on established solid tumors (98). Tessitore et al. found that MG interfered with cell cycle progression, significantly inhibiting the proliferation of rat Yoshida ascites tumor (AH-130) cells, and prolonged the survival of the animals (99). Subsequent studies further confirmed that MG interfered with the growth of Ehrlich ascites tumor cells by inhibiting DNA and protein synthesis (100). MG-induced DNA-DNA polymerase cross-linking severely impedes DNA replication and is one of the key mechanisms for its growth inhibitory effects (43). *In vitro* studies by Thornalley et al. also found that MG induced G1 phase cell cycle arrest in HL60 leukemia cells (IC50 = 238µM) and further triggered apoptosis (DNA fragmentation) at higher concentrations (524 µM) (31, 101). Similarly, Paul-Samojedny et al. demonstrated that MG induced G0 phase arrest and apoptosis in human glioblastoma cells (102). Notably, the toxicity of MG to tumor cells was positively correlated with the rate of cell proliferation and negatively correlated with the degree of differentiation. These studies suggest that one of the key potentials of MG in tumor therapy lies in its ability to inhibit cell cycle progression.

### 4.2 Apoptosis

MG accumulation in cancer cells, especially when GLO1 is inhibited, significantly promotes apoptosis in tumor cells (103). The mechanism of MG-induced apoptosis involves DNA damage, oxidative stress, and the activation of apoptotic signaling pathways.

MG-induced DNA damage plays a central role in initiating apoptosis.MG induces DNA strand breaks through the formation of adducts (e.g., MGdG) and cross-linking structures with DNA, activating the intracellular DNA damage response, and triggering apoptosis by cell cycle arrest when the damage exceeds the cellular ability to repair it (27, 104). The presence of a GLO1 inhibitor significantly enhances the MG-induced DNA damage effects, thereby accelerating apoptosis (27).

Apart from DNA damage, MG-induced apoptosis involves oxidative stress and impairment of mitochondrial function. The apoptosis induced by MG involves the activation of the MAPK pathway and caspase-3 (105). MG can increase the level of ROS or modify mitochondrial proteins, such as the mitochondrial permeability transition pore (PTP), damage the membrane potential, and trigger the mitochondrial-dependent apoptotic pathway (105–107). For example, in L929 fibrosarcoma cells, MG-induced apoptosis was dependent on ROS accumulation (108). Similarly, in triple-negative breast cancer (TNBC) cells and prostate cancer cells, MG promotes activation of the mitochondrial apoptotic pathway by upregulating Bax, downregulating Bcl-2, and inhibiting NF-κB activity (109, 110). Notably, Laga et al. observed that MG inhibits NF-κB activation by directly suppressing the binding of NF-κB p65 to DNA (111). Furthermore, MG amplifies oxidative stress damage and enhances the sensitivity of cancer cells to chemotherapeutic drugs (e.g., cisplatin) by activating multiple signaling pathways, including protein kinase Cδ (PKCδ), JNK, and p38 MAPK (112–114). Notably, cancer cells with weaker antioxidant capacity showed greater sensitivity to MG (115). Moreover, MG-induced p53 nuclear translocation has also been shown to significantly inhibit migration and invasion in hepatocellular carcinoma cells (116). Intracellular calcium overload further exacerbates MG-induced cellular homeostasis disruption and promotes apoptosis (117).

### 4.3 Inhibition of angiogenesis

MG inhibits angiogenesis by disrupting the key molecular interactions required for the formation of new blood vessels in tumors. Specifically, MG modifies the transcriptional coactivator p300 and reduces its ability to bind to HIF-1α, thus limiting the function of HIF-1α to promote angiogenesis under hypoxic conditions and inhibiting nutrient supply to the tumor (118). Furthermore, the modification of extracellular matrix (e.g., type IV collagen) by MG can directly damage endothelial cells, further preventing angiogenesis and even triggering endothelial cell denudation and apoptosis (anoikis), limiting tumor progression (119).

### 4.4 Inhibition of glycolysis

MG blocks tumor cell energy metabolism by directly inhibiting key enzymes of glycolysis (e.g., GAPDH) and interfering with the function of mitochondrial respiratory chain complex I, leading to energy depletion in cancer cells and triggering apoptosis (120–123). For example, in prostate cancer PC-3 cells, MG leads to cell cycle arrest (G1 phase) and significantly inhibits glycolysis, inducing cancer cell death (124).

### 4.5 Immune activation

MG plays a complex role in the tumor immune microenvironment, both by promoting immune escape and, in some cases, by stimulating the body’s anti-tumor immune response. For example, MG treatment in mouse tumor models enhances macrophage phagocytosis and increases splenic CD8⁺ and CD4⁺ T-cell counts, thereby elevating immune cytotoxicity (125, 126). Moreover, MG derivative MG-H1 down-regulates PD-L1 expression in tumor cells in metastatic prostate cancer (mPCa), whereas high expression of GLO1 limits the accumulation of MG-H1 and maintains the expression of PD-L1, which inhibits CD8⁺ T cell function and promotes immune escape in mPCa. This mechanism may explain the resistance of mPCa to immune checkpoint inhibitors (e.g., atezolizumab) (127). Thus, inhibition of GLO1 may enhance the efficacy of immunotherapy against PD-L1 high-expressing tumors, providing a potential therapeutic strategy. In conjunction with Section 3.5, it is clear that MG and its derivatives exhibit a complex dual role in tumor immunotherapy, both driving tumor immune escape and enhancing immune response in specific contexts, with the reasons for this difference still needing to be examined. Therefore, a deeper understanding of their specific mechanisms is crucial for optimizing immunotherapeutic strategies in future studies.

### 4.6 Endoplasmic reticulum stress

MG can modify proteins to make them lose their original structure and function, resulting in the accumulation of misfolded proteins and inducing endoplasmic reticulum (ER) stress response. Glycated proteins lose their normal functions and serve as targets for proteasomal degradation (128). However, covalent modification of the 20S proteasome by MG reduces its degradation activity, leading to persistent activation of the unfolded protein response (UPR), which ultimately triggers apoptosis and inflammatory responses (129, 130). Moreover, MG enhances DNA damage with ROS-induced endoplasmic reticulum stress, which in turn amplifies cGAS-STING pathway activation, leading to greater CD8^+^T cell and NK cell infiltration in the tumor microenvironment, thereby increasing the radiosensitivity of rectal cancer (131). Therefore, MG may be a potential sensitizer and immunomodulator for tumor radiotherapy.

## 5 Targeted therapies for MG

In recent years, targeting MG metabolism by developing MG scavengers and GLO1 modulators has emerged as a promising strategy for cancer therapy. Given the dual role of MG in tumor progression, the rational design of therapeutic strategies must take into account the patient’s metabolic status, disease stage, and tumor type. Targeting high-risk groups with elevated MG levels (e.g., the elderly, obese, and diabetic patients) using MG scavengers or lifestyle modifications to reduce chronic MG stress may be effective in reducing cancer risk. Conversely, inhibition of the MG detoxification pathway may be a proven treatment for tumor cells that have developed into cancer and show increased MG detoxification ability.

### 5.1 MG capturing therapy in the pre-cancerous stage - reducing the risk of cancer

Studies have indicated that MG scavengers such as carnosine can effectively neutralize reactive dicarbonyls and reduce MG stress response (136). In breast cancer cells and ATC cells, MG scavengers (e.g., carnosine, aminoguanidine) as well as GLO1 agonists (e.g., resveratrol) have been shown to reverse the invasive phenotype of cells induced by MG stress (32, 77). In animal models, carnosine has also been found to suppress cancer progression resulting from hyperglycemic conditions, such as pancreatic cancer and breast cancer (74, 75). Furthermore, MG stress is a key driver of drug resistance in cancer cells. For instance, in KRAS-mutant colorectal cancer cells, high glycolytic flux leads to MG accumulation, which activates the Akt survival pathway via the PI3K/mTOR2 axis and Hsp27 regulation, thereby mediating resistance to cetuximab. Specifically, MG-modified Hsp27 is more prone to forming stable functional oligomers with an extended half-life, preventing Akt dephosphorylation and degradation, thereby promoting drug resistance (73). Similar mechanisms are seen in gemcitabine-resistant pancreatic cancer cells, and MG scavengers (metformin, carnosine) are effective in reversing these resistances (95, 96). This suggests that MG scavengers and GLO1 agonists are not only indicated for cancer prevention but may also improve the sensitivity of chemotherapy, especially in high-risk groups with metabolic abnormalities (**Table 3**).

**Table 3:** The therapeutic role of MG scavengers in cancer.

MG scavenge	Cancer Categories	Model	Dosage	Laboratory Findings	References
Metformin/aminoguanidine	PC	T3M4	10mM	HSF1, HSP27, HSP90↓ Suppress the proliferation of gemcitabine-resistant cells.	(95)
Carnosine		MIA PaCa-2	MG:200uM+Carnosine: 20 mM	▲ YAP↓ ▲ Cell proliferation↓	(75)
Carnosine/aminoguanidine	BC	shGLO1-MDA-MB-231/468;MCF7	10mM	Decrease the migratory and invasive capabilities induced by MG stress.	(77)
Carnosine		shGLO1-MDA-MB-231 mice	100mg/kg Intraperitoneal	Pulmonary tumor burden and Metastatic foci↓	
Carnosine	CRC	KRAS^G12V- SW48 mice	Carnosine: 100 mg/kg+ Cetuximab: 0.5mg Intraperitoneal	▲ PARP, caspase-3↑ ▲ Cell apoptosis↑ Tumor growth↓	(96)
		KRAS^G12V- SW48	Carnosine: 100mM+ Cetuximab:30ug/ml		
Aminoguanidine/resveratrol	ATC	AL62/8505C	AG: 1mM/ Resveratrol: 50uM	Attenuate the migratory and invasive capacity of cells.	(32)

↑: High expression/promotion; ↓: Low expression/inhibition.

Notably, diet and lifestyle can have a significant impact on the levels of MG and its derived AGEs. Both high-fat and high-sugar diets, as well as smoking, increase the production of AGEs (7, 65). Specific cooking methods (e.g., grilling, deep-frying) also increase the level of AGEs in food (137). Various dietary strategies have been proposed to reduce the load of MG/AGEs. For example, dietary intake of quercetin and genistein was effective in reducing MG levels in plasma and tissues in mice (138, 139). A randomized controlled study that included 540 subjects showed that following a Mediterranean diet based on vegetables, fruits, fish, whole grains, legumes, and olive oil was effective in reducing serum MG and AGEs levels in humans (140). Therefore, the intake of MG/AGEs can be reduced through the following dietary interventions: (I) Reducing the intake of exogenous MG/AGEs, such as avoiding high temperature processing (barbecuing, frying, baking), recommending low temperature cooking methods such as steaming, boiling, stewing, and reducing the intake of foods high in sugar and fat (such as processed foods, red meat, refined carbohydrates); (II) Increasing the intake of MG scavengers and antioxidants, such as chicken, beef and fish rich in carnosine to directly remove MG and inhibit the generation of AGEs (141); Antioxidant foods such as vegetables, fruits, beans and olive oil, which are rich in polyphenols and dietary fiber, can effectively reduce the oxidative stress and inflammatory response induced by MG (142), all of which contribute to reducing the risk of cancer and improving the prognosis of cancer patients.

### 5.2 MG toxic therapy in the cancer stages - enhancing treatment sensitivity

#### 5.2.1 GLO1 overexpression—protective adaptive mechanisms in cancer cells

Similar to Darwinian evolutionary theory, highly glycolysis-dependent cancer cells have evolved adaptive protective mechanisms under the selective pressure of MG, the most critical of which is the high expression of GLO1 (143). GLO1 overexpression endows cancer cells with stronger MG detoxification ability, prevents them from MG-induced apoptosis, and promotes tumor invasion and development of drug resistance (9, 144) (a discussion of the toxic killing effects of MG on cancer cells can be found in Section 4). Currently, high GLO1 expression has been confirmed in various malignant tumors, including gastric cancer, pancreatic cancer, endometrial cancer, hepatocellular carcinoma, and prostate cancer, and is closely associated with tumor invasion, metastasis, and chemotherapy resistance (143, 145–147). In breast cancer, lung cancer, bladder cancer, and colon cancer, GLO1 gene amplification was significantly associated with poor survival outcomes, suggesting that GLO1 could be used as a biomarker for poor prognosis (148, 149). At the same time, the expression of GLO1 showed variability in different types of the same tumor. For example, proteomic studies of breast cancer revealed that overexpression of GLO1 was significantly associated with the grading of breast cancer (150). In bladder cancer, superficial bladder cancer (SBC) exhibited higher GLO1 activity than invasive bladder cancer (IBC) (151). This finding suggests that GLO1 can be used as a marker to differentiate tumor grading and typing, which in turn improves accuracy and reliability in clinical diagnosis. Therefore, GLO1 is not only suitable as a predictive marker for tumor progression and therapeutic resistance, but also has potential clinical applications: by quantitatively detecting the expression level of GLO1 in cancer tissues or circulating tumor cells, the response or resistance risk of patients to conventional chemotherapeutic drugs can be assessed before clinical treatment, and then optimize personalized treatment plans. In addition, GLO1 may also be an important therapeutic target by inhibiting GLO1 activity and inducing MG toxicity to selectively kill cancer cells, thus providing a new strategy for the treatment of drug-resistant cancers. (**Table 4**).

**Table 4:** The therapeutic effects of GLO1 inhibitors in cancer.

GLO1 inhibitor	Cancer Categories	Model	Dosage	Laboratory Findings	References
BBGC	Leukemia	HL60	IC50 = 4.23 ± 0.01uM	Suppress cell growth, inhibit DNA synthesis, and induce cell apoptosis.	(103)
	MOL/CML	UK711/ K562	12.5uM	▲ caspase↑, Cell apoptosis↑ ▲ Augment the therapeutic sensitivity of drug-resistant cells.	(144)
	CML	HA-K562/KCL22	21.6uM/40.7uM	Cell apoptosis↑	(154)
		HA-K562 mice	10mg/kg Intraperitoneal	The survival duration of mice↑	
	NB	SH-SY5Y	10uM	▲ MG, JNK, p38 MAPK, caspase-3↑ ▲ Cell viability↓	(166)
	CML	HA-CML mice	10mg/kg, gavage	▲ Cell apoptosis↑ ▲ The survival duration of mice↑	(154)
EP	AMOL	THP-1	IC50 = 1.4 ± 0.2mM	▲ LDH, ATP↓ ▲ Cell necrosis	(155)
Curcumin	BC	MCF-7	24h IC50 = 24.5uM 48h IC50 = 11.4uM	▲ Mitochondrial dysfunction↑ ▲ Cell proliferation↓	(132)
OT55	HCC	SMMC-7721	1.56-50uM	MG, Cell apoptosis↑	(164)
		mice	10mg/kg, Intraperitoneal	▲ MG, STAT1, p53, Bax↑ ▲ c-Myc, Bcl-2↓ ▲ Cell apoptosis↑ Tumor growth↓	

↑: High expression/promotion; ↓: Low expression/inhibition.

#### 5.2.2 Targeting GLO1 therapy—new strategies to improve treatment sensitivity

Therapies targeting GLO1 offer a novel strategy for cancer therapy. Preliminary studies have shown that targeting GLO1 by small molecule inhibitors such as S-p-bromobenzylglutathione cyclopentyl diester (BBGC) significantly inhibits the growth and induces apoptosis of leukemia and other tumor cells, while reversing the resistance of cancer cells to chemotherapeutic drugs (103, 144, 152–154). Furthermore, the competitive GLO1 inhibitor ethyl pyruvate (EP) showed specific antitumor activity in leukemia cell lines, mainly by depleting cancer cell ATP reserves without impairing normal cell function (155). The anticancer effects of methotrexate and troglitazone are also partially dependent on GLO1 activity inhibition, further highlighting the potential of GLO1 as a predictor of treatment response (156, 157).

In breast cancer models, MG induction or GLO1 inhibition can achieve significant anticancer effects by activating the MAPK signaling pathway and decreasing the expression of anti-apoptotic proteins (e.g., Bcl-2, MMP-9) (133). Of interest, GLO1 inhibitors have been shown to reverse the sensitivity of tamoxifen-resistant breast cancer cells, revealing that GLO1 may be a biomarker for predicting the efficacy of endocrine therapies and a target for overcoming treatment resistance (158). Curcumin, as a natural inhibitor of GLO1 activity, inhibits the proliferation of breast cancer cells by inducing mitochondrial dysfunction (132). Moreover, non-pharmacological interventions, like peritumoral electroacupuncture, have also been demonstrated to increase tumor sensitivity to chemotherapeutic agents by inhibiting GLO1, further strengthening the potential of GLO1 as a targeted therapeutic indicator (159).

In lung cancer, inhibition of GLO1 expression activity leads to MG accumulation, which can significantly inhibit tumor growth and induce cell apoptosis (135, 160). Similarly, GLO1 inhibitors have been shown to limit tumor progression and increase the sensitivity of conventional anticancer therapeutic agents in colorectal cancer (134, 161), melanoma, hepatocellular carcinoma (147, 162), and gastric cancer (163). For instance, 3,3’-[3-(5-chloro-2–35 hydroxyphenyl)-3-oxopropane-1,1-diyl]bis(4-hydroxycoumarin) (OT-55) inhibits the GLO1 activity, induces MG accumulation and triggers apoptosis of liver cancer cells (164). Other GLO1 inhibitors, such as BBGC, TER 117, and shikonin, have also shown synergistic chemotherapy sensitization effects when combined with anticancer drugs (165–167).

In summary, targeted GLO1 inhibition therapy can effectively improve the sensitivity of tumor treatment by selectively utilizing the accumulation of MG toxicity in cancer cells, especially showing outstanding value in the treatment of drug-resistant tumors. Meanwhile, as a potential biomarker of prognosis and treatment response, GLO1 can assist clinical decision-making and optimize treatment options, thereby promoting the further development of precision medicine in oncology.

## 6 Conclusion and perspective

The dual role of MG in cancer reveals its intricate and diverse functions in cellular metabolism and tumor progression. On the one hand, as a toxic by-product of glycolysis, high concentrations of MG exhibit significant anti-tumor effects by inducing apoptosis, DNA damage, energy metabolism disruption, and endoplasmic reticulum stress. On the other hand, at lower concentrations or under long-term chronic exposure conditions, MG promotes cancer cell proliferation, invasion, and metastasis through glycation modification, AGEs-RAGE signaling pathway, and immune microenvironment remodeling, driving tumor progression. This apparent dose-dependent “double-edged sword” characteristic, i.e., hormesis, opens up new research ideas and therapeutic possibilities in the field of tumor metabolism and tumor microenvironment research.

It is important to note that differences in the susceptibility of different cancer cells and tumor subtypes to MG effects may depend on differences in intracellular MG cumulative load, MG detoxification ability (e.g., GLO1 activity), and intracellular redox homeostasis. However, the specific threshold concentration of MG has not yet been clarified, and how different exposure frequencies and durations affect the survival, apoptosis, or transformation potential of tumor cells remains an important question to be addressed. In addition, the performance of MG-regulated dose thresholds *in vivo* tumor models and clinical patients has not been systematically investigated. Therefore, future studies need to further clarify the following key issues: (I) Determine the safety and toxicity dose thresholds of MG in different cancer types, and clarify at what concentration and exposure time the effect of MG shifts from cancer-promoting to cancer-suppressing, which would provide a precise biological basis for the dose-dependent effect of MG; (II) Systematically assess the variability of GLO1 activity and MG metabolic load in different tumor cell types and tumor microenvironments, and to gain a deeper understanding of how cellular stress induced by MG exposure affects the survival, apoptosis, and invasive capacity of cancer cells, thus providing theoretical support for individualized therapy; (III) Explore the synergistic mechanisms and combined application strategies of MG modulation with existing clinical therapies (e.g., chemotherapy, radiotherapy, immunotherapy), especially in the context of therapeutic drug resistance to clarify the specific mechanisms and clinical significance of MG in overcoming drug resistance; (IV) Elucidate the exact mechanism of MG stress, oxidative stress and chronic inflammation cross-linking network in the construction of tumor microenvironment, and to identify the key nodes of MG-related signaling pathways in the dynamic evolution of the tumor microenvironment, so as to develop effective intervention strategies.

In terms of clinical application, targeting the MG metabolic pathway provides a dual therapeutic strategy: first, for tumors with high glycolytic activity, by inhibiting GLO1 activity to selectively elevate MG levels in cancer cells and enhance the sensitivity of tumor cells to therapy; and second, for high-risk populations (e.g., diabetes, obesity, and the elderly), by reducing MG stress through MG scavengers or GLO1 agonists to prevent cancer progression. However, how to accurately control MG levels to maximize efficacy and minimize toxicity in the course of clinical treatment and cancer prevention remains an important challenge.

In conclusion, the dose-dependent threshold of MG effects, individual variability, and its synergism with cancer therapeutics must be clarified in future cellular, animal model, and clinical studies. This not only has important theoretical significance for understanding the role of MG in tumor biology, but also provides a broad prospect for the development of precise and individualized cancer therapies based on the MG metabolic pathway.
